# Advanced Molecular and Microscopic Diagnostics Suggest Congenital *Borrelia* Transmission: A Case Report

**DOI:** 10.3390/microorganisms14020406

**Published:** 2026-02-09

**Authors:** Lynne T. Bemis, Maryna Golovchenko, Marna E. Ericson, Md. Hasibul Haque, Vett Lloyd, Natalie Rudenko

**Affiliations:** 1 Department of Biomedical Sciences, Duluth Campus, Medical School, University of Minnesota, Duluth, MN 55812, USA; ltbemis@d.umn.edu; 2 Biology Centre Czech Academy of Sciences, Institute of Parasitology, 370 05 Ceske Budejovice, Czech Republic; marina@paru.cas.cz; 3 Department of Dermatology, University of Minnesota, Minneapolis, MN 55455, USA; erics004@umn.edu; 4 Department of Biology, Mount Alison University, Sackville, NB E4L 1G7, Canada; mhaque@mta.ca (M.H.H.); vlloyd@mta.ca (V.L.)

**Keywords:** *Borrelia burgdorferi*, Lyme disease, non-vector routes of *Borrelia* transmission, congenital transmission of *Borrelia*, vector-borne and vertically transmitted disease

## Abstract

Lyme disease is by far the most common arthropod-borne disease in the Northern Hemisphere. It is caused by certain *Borrelia* species that are primarily transmitted to hosts by Ixodid ticks; however, transplacental transmission of the spirochete in both animals and humans has been reported. Here, we report imaging of intact spirochetes in an archived placental tissue sample that is immunoreactive to *Borrelia* antibodies and from which *Borrelia* DNA was isolated. Both mother and child showed evidence of seroreactivity to *Borrelia* spp. in the years following the child’s birth, although neither individual tested positive by the conventional two-tiered serological algorithm. Cultivation of viable spirochetes from a vaginal swab of the mother and from the urine of the child some years later supports the possibility of vector-free transmission of *Borrelia* from mother to child. By amplifying several genomic loci from the DNA of cultured and non-cultured *Borrelia* from blood and body fluid samples of the mother and child, the *Borrelia* in both were identified as the same species, *Borrelia burgdorferi* sensu stricto, a strain specific to North America.

## 1. Introduction

The causative agent of Lyme disease (LD), *Borrelia burgdorferi*, was first reported in 1982 [1,2]. When the connection between LD, ticks and *Borrelia* was first discovered, tick bites were recognized as the primary mode of spirochete transmission [1,2,3] from infected ticks to vertebrate hosts, including humans. Transmission of tick-borne pathogens is clearly via ticks. Yet, this does not exclude the existence of other modes of infection. Maternal–fetal transmission of LD spirochetes has been documented over the last 40 years since the pioneering case report by Schlesinger et al. (1985) [4], followed by other findings confirming the ability of the spirochete to cross the human placenta and infect the fetus [2,4,5,6,7,8,9,10]. Thus, the potential for human-to-human transmission of *B. burgdorferi* spirochetes in the absence of a tick vector, specifically in utero transmission, was acknowledged, although it was considered rare [11].

The possibility of transmission of the LD spirochete without the tick vector was raised as early as 1986, when Burgess and colleagues published their findings of direct contact transmission of *B. burgdorferi* between infected and uninfected wild mice, *Peromyscus leucopus* and *P. maniculatus*. Uninfected mice in contact with infected mice, of both species, developed antibodies to *B. burgdorferi* by day 14 after exposure to infected cage-mates. Further, spirochetes were recovered from the blood of one contact-exposed *P. maniculatus* 42 days after initial contact [12]. Subsequent studies by Wright and Nielsen demonstrated the susceptibility of mice to oral infection with *B. burgdorferi* and transmission of spirochetes from infected males to uninfected females by direct contact [13]. Canine models added further evidence for the possibility of vector-free transmission of *Borrelia*; an uninfected female dog seroconverted from negative to positive after natural breeding with an experimentally infected male dog. *Borrelia* DNA was detected in tissues of fetuses from the following pregnancy [14]. More evidence for in utero transmission of *Borrelia* in animals was reported: in mice and rodents [15,16,17,18,19,20], dairy cows and horses [21,22,23], vixens, dogs and coyotes [14,24,25]. Direct detection of *Borrelia* in tissues was demonstrated by various methods including immunofluorescent staining, PCR and culture.

In later years, additional reports documented cases of possible congenital transmission [26,27] and placental infection [28,29,30,31] by the causative agent of LD in both North America and Europe. In addition to human data, experimental animal studies investigating vertical transmission of *B. burgdorferi* produced mixed results, with some reporting its occurrence [14,15,16,17,24] while others found no such transmission [13,32,33,34]. Studies involving naturally infected domestic animals [21,22,23] and wildlife populations [14,18,19,20,25] also reported evidence for vertical transmission of *B. burgdorferi*.

In addition, *Borrelia* has been identified in both human and animal milk. *Borrelia* DNA in cow’s milk and cow’s colostrum was detected by PCR [23,35]. Furthermore, cultivation of spirochetes from cow colostrum [21], transmission through milk in mice [17], and a case of a cat seroconverting after being fed milk from a *Borrelia*-infected cow [36] support this route of infection. In humans, at least two cases are known in which the presence of *Borrelia* DNA in milk was detected by PCR in lactating women with erythema migrans who were not treated with antibiotics. Live spirochetes were cultured from a skin biopsy from one of the tested women [37].

Currently, the US Centers for Disease Control and Prevention (CDC) reports approximately half a million LD cases annually in the USA alone [38]. A revised estimate of LD cases in Europe is almost 850,000 per year [39]. The risk of in utero transmission of *B. burgdorferi* is recognized by North American public health-related institutions [40,41,42]; however, there are no standardized diagnostic guidelines for congenital LD. Even though the acknowledgment of the existence of minor or alternative modes of spirochete transmission is slow and still raises doubts, they are worthy of consideration. The question of vertical transmission of the LD spirochetes (any transmission from mother to offspring) is a topic that requires immediate attention as, in humans, it is linked to “…adverse pregnancy outcomes including neonatal death” [2]. This topic deserves urgent attention since confirmation of transmission from mother to child elevates the complexity of LD diagnosis and treatment.

In this report we present a case of possible vertical transmission of *B. burgdorferi*, detected using advanced methods including indirect immunofluorescence, PCR, and bacterial culture, confirmed by different laboratories.

## 2. Materials and Methods

### 2.1. Research Subjects and Informed Consent

The adult subject (age 39) consented to participate in a research study for LD testing for both herself and her child (age 6). The adult subject provided relevant medical records for this study. Written consent was obtained, and this study was approved by the Mount Allison Research Ethics Board 2016-042/101796.

### 2.2. Culture of Borrelia from Participants’ Samples

All samples were cultured in Barbour–Stoner–Kelly H (BSK-H) complete medium containing 6% rabbit serum (Dalynn Biologicals, Burlington, ON, Canada) with the addition of the following antibiotics as described by Berthold [43]: phosphomycin (0.002 mg/mL; Sigma-Aldrich), rifampicin (0.005 mg/mL; Sigma-Aldrich, St. Louis, MO, USA), and amphotericin B (0.25 µg/mL; Sigma-Aldrich). Samples were collected from different body fluids and inoculated as follows:

A. Urine: Midstream urine samples were collected in a sterile container, and then approximately 1 mL was immediately introduced into BSK-H medium with a sterile pipette by the adult subject.

B. Periodontal/Mouth swab: Periodontal swabs were collected using sterile cotton-tipped swabs, which were immediately inoculated into BSK-H medium.

C. Vaginal fluid: Vaginal fluid was self-collected by the adult subject by swabbing the inside of the vagina with a sterile cotton-tipped swab, which was immediately introduced into BSK-H medium.

All supplies and instructions were provided to the adult participant by the Canadian research laboratory. Collection and inoculation of culture tubes were performed at the participant’s home and shipped at room temperature to the Canadian laboratory. Cultures were incubated at 34 °C for 6 weeks, after which microscopic and molecular analyses were performed. To avoid contamination, all procedures were carried out in a Biological Safety Cabinet LabGard Class II, Type A2 (NuAire, Plymouth, MN, USA). The Biological Safety Cabinet was thoroughly cleaned with ethanol and sterilized with UV radiation before each sample was processed. Only one tube was open at a time.

### 2.3. DNA Extraction and PCR Analyses of Cultured and Uncultured Samples

DNA was extracted from the cultures using the Aquaplasmid kit (MultiTarget Pharmaceuticals, Colorado Springs, CO, USA) or the DNeasy Blood and Tissue Kit (Qiagen, Valencia, CA, USA) according to the manufacturer’s recommendations (see Supplementary Materials). Total DNA purified from both cultured and uncultured *Borrelia* was used as the template in multiple PCR reactions targeting different genomic loci of *Borrelia* spp.: *flaB*, *ospA*, *ospC*, 16S-23S ITR, *p13*, *p66*, *recG*, *rplB* and *uvrA*. Conditions for amplification of each genomic locus are described in the Supplementary Materials. Specific PCR primers are listed in Tables S1–S3. DNA extracted from archival placental blocks was amplified using internal primers for part of the gene encoding *flaB* (Supplementary Materials). Amplicons obtained by the Canadian and Czech Republic laboratories were sequenced in both directions using the same primers as for PCR. The amplicons from the USA laboratory were cloned into the pCR2.1 TOPO vector for sequencing (K4560-01, Life Technologies, Carlsbad, CA, USA) [44]. The negative control for PCR included water only and did not provide amplicons, and the positive PCR products were sequence-confirmed. In the Canadian laboratory, no pure *Borrelia* cultures or clinical cultures other than those from this study were in the laboratory at the time of this work. The laboratory had tick samples from North America, from which DNA was being extracted, in the lab at the time of this study. However, DNA extraction, PCR amplification and gel electrophoresis were all done in different laboratory spaces with isolated air flows to reduce contamination, and human and tick DNA extractions were performed in different rooms. In all cases, no-template negative controls (NTCs) remained negative. The European laboratory did not possess any other growing cultures, clinical samples, or DNA from *B. burgdorferi* s.s. species before, during, or after the proposed study. Negative controls were always run separately and did not produce any amplicons. As the positive control, DNA from *B. carolinensis* was used by the European laboratory, and the resulting amplicons were always sequence confirmed. In the US laboratory the only formalin-fixed samples processed during this study were from this patient; no *Borrelia* spp. were growing in the lab at that time. Positive controls were from *Borrelia* DNA extracted from cultures of North American *B. burgdorferi* s.s.; however, all of the NTCs confirmed the absence of contamination, and *flaB* had not previously been amplified in that laboratory.

### 2.4. Multi-Staining and Confocal Imaging of Tissues

Archival tissue blocks were obtained from Trillium Health Partners, Mississauga Hospital, Mississauga, Ontario. Tissue blocks from (A) umbilical cord, (B) one from placental membranes and (C, D) two placental tissue blocks (Supplemental Figure S1) were sectioned at 5 microns, and staining procedures followed previously described methods [45]. Briefly, tissue sections were deparaffinized and incubated with blocking serum (normal donkey 017-000-121, Jackson Immuno Research Laboratories, West Grove, PA, USA), a primary anti-*B. burgdorferi* antibody tagged with FITC at 1:100, made in rabbit (MyBiosource, MBS324026, San Diego, CA, USA) and the nuclear dye, 4′,6-diamidino-2-phenylindole (DAPI) at 3 µmol (Thermo Fisher, Waltham, MA, USA). Another set of deparaffinized sections was used as no primary antibody controls for each tissue block. Remaining tissue sections were used for DNA extraction as described in Section 2.5. Stained samples were mounted in Vectashield Plus Mounting Medium (Vector Laboratories, H-1900, Newark, CA, USA). Images were captured by single-photon laser confocal microscopy.

### 2.5. DNA Isolation from Formalin Fixed Paraffin-Embedded Tissue Blocks

The remaining tissue from the imaging experiment was used for further DNA extraction and PCR analyses utilizing the DNA portion of the AllPrep DNA/RNA FFPE kit (Qiagen, No. 80234, Valencia, CA, USA). The brief step-by-step procedure for deparaffinization of embedded tissues is provided in the Supplementary Materials. Bacterial DNA found in the flow-through after the first spin of the column was used for further PCR analysis (see Supplementary Materials).

### 2.6. Processing of Ungrown Borrelia from the Blood Samples

Plasma fractions obtained from blood samples of child and adult subjects in Canada were shipped to the European laboratory. Upon arrival 48 h later, having been shipped under uncontrolled temperature conditions, the samples were placed at +33 °C to facilitate the growth of live spirochetes. One week after arrival, dark field microscopy was conducted regularly. Contaminated cultures were filtered through a 0.2 µm syringe PES filter, and the media were replaced. HiMedia 100× Antibiotic Mixture (HiMedia, Mumbai, India) was added to the cultures to achieve final concentrations of 0.002 mg/mL phosphomycin, 0.005 mg/mL rifampicin and 0.25 µg/mL amphotericin [43]. After three weeks of cultivation the cultures were centrifuged at 2000 rpm for 30 min, and the media were replaced. After a further three weeks of cultivation, when active growth of spirochetes was not apparent, all cultures were centrifuged at 13,200 rpm for 20 min, and the pellet was used for total DNA purification (see Supplementary Materials). PCR analyses were conducted using published PCR primers and strictly followed the conditions established for each primer set (see Supplementary Materials) [46,47].

## 3. Case Presentation and Results

The adult subject was a resident of a Lyme disease risk area in southern Ontario, Canada. She had lived in Singapore, Malaysia, and Kenya. At age 32, during the first trimester of her fourth pregnancy, the mother experienced fever and flu-like symptoms while living in Kenya. Malaria was suspected but excluded through repeated blood smears. At birth, pathological examination of the placenta, conducted due to the first trimester presumed infection, revealed a term placenta with no significant pathological abnormality, an umbilical cord with three vessels, and no evidence of inflammation. Following the birth, the adult female experienced ongoing and unrelenting fatigue and migratory pain. At age 38, Canadian Public Health Lyme disease serology showed reactivity on the enzyme immunoassay (EIA) but negative Western blots (IgM and IgG). However, a EUROIMMUN *Borrelia* line blot was positive (NML, Winnipeg, MB, Canada). The adult female was subsequently clinically diagnosed with Lyme disease by an infectious disease physician in Ontario, Canada, and received a ten-week course of intravenous ceftriaxone. Additional tests for Lyme disease showed a positive T-lymphocyte activation assay against *Borrelia* spp. (Infectolab, Augsburg, Germany) and a CDC-positive Lyme IgM Western blot (IgeneX, Palo Alto, CA, USA). In 2023, archived placental samples were requested from the delivery hospital for research purposes.

The child subject was a female resident of a Lyme disease risk area in southern Ontario, Canada. She lived continuously in Canada but was in utero in Kenya. She was delivered vaginally at 37 weeks of gestation, weighing 7 Ib 2 oz. She subsequently developed hyperbilirubinemia and was hospitalized overnight for phototherapy. At 11 weeks of age, she developed a high fever and was assessed in the Emergency Department, although routine testing did not identify a cause. Early childhood was characterized by cyclical fevers and cyclical vomiting. At age 4, symptoms of unexplained pain and hypersensitivity to light and sound developed. At that time an IgeneX Lyme IgG Western blot (Palo Alto, CA, USA) revealed positive bands for 31, 34, 39, 41, and 58 kDa, while an IgM Western blot revealed the band for 41 was positive and an indeterminate band for 39 kDa. At age 5, in early winter, following another febrile episode, multiple transient erythematous patches developed. Serological testing for Lyme disease was performed and reported as negative (Public Health Ontario, Toronto, ON, Canada). At age 5 the child was diagnosed with LD and *Bartonella henselae* infection by a physician in the USA, and antibiotic treatment was initiated. A *Borrelia* ELIspot LFA-1 (Arminlabs, Augsburg, Germany) was positive. Serology for *Bartonella henselae* (IgG) was positive (IGeneX, Palo Alto, CA, USA) as well. Later, at the age of 7, a Lyme EIA was positive while Lyme IgG and IgM Western blots were negative (Public Health Ontario, Canada). Additional serological testing performed at the National Microbiology Laboratory, Canada, against *Borrelia afzelii* and *Borrelia garinii* was negative. No tick bite had been noted during childhood.

Body fluids were collected from the adult and child subjects (Table 1) and analyzed in three geographically distant laboratories at different times. DNAs extracted from cultured blood samples from both subjects were analyzed in the European laboratory. Cultures were obtained from urine and, for the adult subject, vaginal and periodontal swabs, and were analyzed in the Canadian laboratory. Placental tissues from formalin-fixed, paraffin-embedded (FFPE) archived samples were analyzed in the USA laboratory.

Multiple sets of primers, targeting the *p66*, *ospC*, *flaB*, *recG*, *rplB* and *uvrA* genes were used (see Supplementary Materials, Table S2). For the adult subject, fragments of the genes encoding *p66* and *flaB* were obtained. In the case of the child subject samples, amplicons were obtained for the *p66*, *flaB*, *ospC*, *rplB* and *uvrA* genes (Table 1). Alignment of partial sequences of the *p66* gene (236 bp) showed 99.58% identity at the DNA level between the adult and child subjects, with a single silent A–G substitution; the identity of the translated sequences was 100% (Supplementary Materials). Amplification of the partial *flaB* gene from the adult and child subjects resulted in two identical sequences. Analysis of a partial *ospC* sequence (620 bp) obtained from the child’s sample revealed 100% identity to the sequences of North American *B. burgdorferi* s.s. strains belonging to the *ospC* type A group (Supplementary Materials), the most widely distributed type of *ospC* gene in the New World. Analyses of partial sequences of the *rplB* gene (712 bp) and *uvrA* gene (679 bp) obtained from the child’s sample (Supplementary Materials) confirmed, using the PubMLST database (www.pubmlst.org), that the *B. burgdorferi* s.s. strain belonged to the North American *B. burgdorferi* s.s. group.

Although the presence of *Borrelia* DNA in the blood of both the adult and child subjects was confirmed by amplification of multiple *Borrelia* genomic loci, cultures of viable *Borrelia* from this set of blood samples were negative. However, spirochetes were successfully cultured from body fluids of both subjects in BSK-H media that had not been exposed to international shipping. Following culture in the Canadian laboratory, molecular analysis performed on all samples identified *B. burgdorferi* sensu stricto. For both subjects, amplification of the *B. burgdorferi ospA* gene was obtained for at least one sample (Table 1; Supplementary Materials). The amplicon of the expected size, 350 bp, was obtained from DNA from the vaginal swab-seeded cultures of the adult and urine-seeded cultures for the child. Purified DNA fragments were sequenced and showed 100% identity to the *ospA* fragments of a wide variety of North American *B. burgdorferi* s.s. strains (Supplementary Materials). PCR targeting the *flaB* gene and the 16S–23S internal transcribed region did not produce amplicons. The culture results demonstrate live *Borrelia* in both the mother and child at ages 39 and 5, respectively, when the samples for culture were donated, but do not provide information on when the infection might have occurred.

To gain insight into possible transplacental transmission, four archival formalin-fixed paraffin-embedded samples were obtained from the hospital where the child was born. The four samples included a section of the umbilical cord, membranes and two sections of placental tissue (Supplemental Figure S1).

Following deparaffinization and DNA isolation, PCR amplification of partial *flaB* and *p13* genes and sequencing of the amplicons confirmed the presence of *Borrelia* species in sections of the placental tissue. The *flaB* amplicons showed high similarity to North American *B. burgdorferi* s.s. while the *p13* amplicon is conserved across numerous *Borrelia* species.

**Figure 1:**
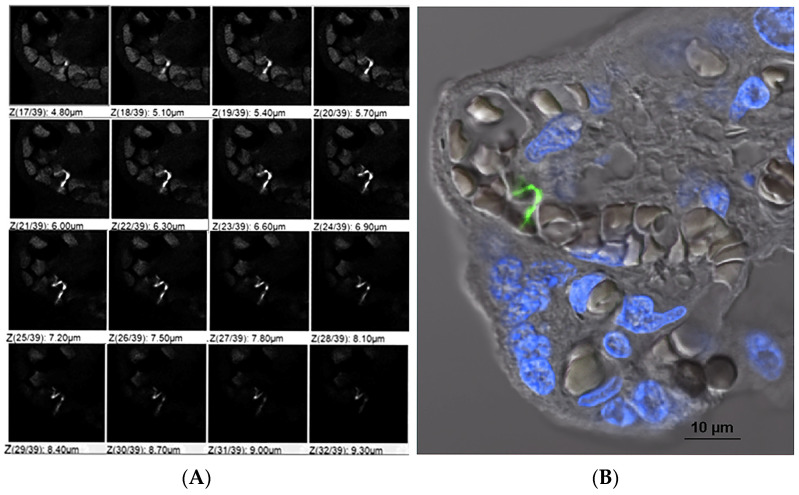
Immunostaining of *B. burgdorferi* in placental membranes. A formalin-fixed paraffin-embedded (FFPE) placental membrane sample was taken at birth and stained for immunoreactive (ir)-*B. burgdorferi* fourteen years later (Block B, Supplemental Files). The section was imaged using single-photon laser scanning microscopy at 0.3 micron intervals in the z-plane. (**A**) Sixteen individual single-slice z-steps, 0.3 micron increments, reveal the three-dimensional nature of the pleomorphic ir-*B. burgdorferi* spirochete (white). (**B**) The entire three-dimensional z-stack of this section stained with the nuclear dye DAPI (blue), ir-*B. burgdorferi* (green) and transmitted light (gray). The three-dimensional Z-stack projection consists of 39 sections, each at 0.3 microns, acquired using a Nikon Ti2 confocal microscope (PLAN APO λD 40× objective, Tokyo, Japan). Measurements indicate the pleomorphic bacterium is approximately 14 microns in length (scale bar, 10 microns).

**Figure 2:**
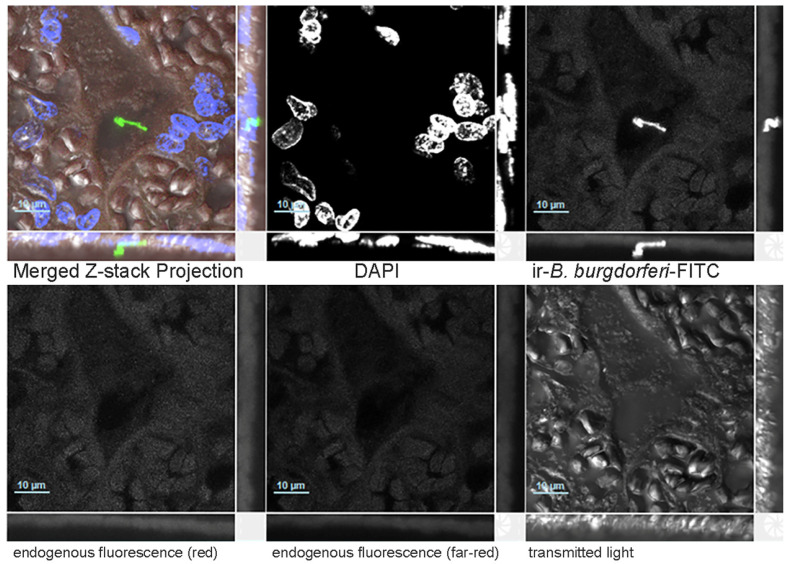
Immunostaining of *B. burgdorferi* in placental sample. A FFPE placental tissue sample from the same birth (Block D supplemental file) was similarly stained and imaged using confocal microscopy. The entire Z-stack projection is shown in split channels used to generate the merged images. The merged image includes all five channels: DAPI staining (blue), ir-*B. burgdorferi* (green), endogenous tissue staining, and transmitted light. The Z-stack projection consists of 35 sections, each at 0.3 microns, acquired with a Nikon Ti2 confocal microscope (PLAN APO λD 40× objective, scale bar 10 microns).

The presence of spirochaetes in the placental tissue was further verified by confocal microscopy of immunoreactive *B. burgdorferi* in the placental tissue (Figure 1 and Figure 2).

In summary, DNA from a *B. burgdorferi* sensu stricto strain highly similar to the North American group of Lyme disease spirochetes was detected in various samples from the mother and child subjects in three independent laboratories. The group of different genomic loci targeted included genes encoding *flaB*, *ospA*, *ospC*, *p13*, *recG*, *rplB*, *uvrA* and 16S–23S internal transcribed region. Not all amplifications were successful. These findings are supported by direct visualization of an immunostained spirochete in archival placental tissue and the cultivation of viable spirochetes from the body fluids of both subjects.

## 4. Discussion

We report the detection of morphologically intact *Borrelia* spirochete in an archival placental tissue sample collected at childbirth, using indirect immunofluorescence microscopy. Analysis of DNA extracted from the same archival samples confirmed the presence of *B. burgdorferi* sensu stricto. Independent molecular analyses of live spirochetes, which were successfully cultured years later from the mother’s vaginal swab and the child’s urine, confirmed the identification of the detected spirochetes as *B. burgdorferi* sensu stricto. Additionally, amplification of multiple genomic loci from blood samples of both the adult and child subjects identified the *B. burgdorferi* sensu stricto strain specific to North America. Our finding of viable *Borrelia* in vaginal swabs is consistent with the work of others. The culture of *Borrelia* was reported from human semen and vaginal secretions [48], and spirochete DNA was amplified by PCR from 11 out of 13 patients diagnosed with LD, and motile spirochetes were observed in genital culture concentrates from 12 of 13 LD patients by dark-field microscopy. In this case study, we do not have access to data that would indicate the origin of *Borrelia* infection in the adult participant. The sites of residence of the adult subject includes an endemic area of Canada, Singapore and Malaysia where the LD cases caused by *Borrelia afzelii* (vectored by *Ixodes granulatus*) have been described [49] and Kenya, where LD cases are quite rare, but the presence of *Borrelia garinii* (the major cause of neuroborreliosis in Europe) introduced there by migratory birds from northern Europe has been confirmed [50]. The results of this study with multiple molecular and microbiological methods applied by different laboratories, along with imaging of immunoreactive *Borrelia*, strongly indicate the existence of a *B. burgdorferi* sensu stricto strain specific to North America at the child’s birth.

Various case reports of maternal infection with subsequent vertical transmission have been published highlighting the significant complexity and diversity of clinical disease presentations. These reports suggest a possibility, even if infrequent, of non-tick-vectored routes of pathogen transmission between humans. Most published reports related to vertical transmission of LD spirochetes were conducted in North America and deal with transmission of *B. burgdorferi* sensu stricto. Epidemiological studies offer limited information to support or refute any association between maternal infection, congenital malformations and adverse outcomes [51]. A heterogeneous range of adverse pregnancy outcomes have been reported with gestational LD. Adverse outcomes include premature delivery, miscarriage, stillbirth and neonatal death [52,53,54]. A diversity of clinical manifestations have been reported in the newborn, ranging from low birth weight, hyperbilirubinemia, hypotonia, cortical blindness, developmental delay, cardiac and urinary tract defects, syndactyly, respiratory distress, and newborn rash [52,53,54]. A case of transplacental transmission of LD was reported whereby the mother gave birth to an initially normal appearing baby who died 8 days later. Authors reported culturing of *B. burgdorferi* from frontal cortex and silver staining of brain and heart, however the mother was seronegative for LD [8]. That a uniform pattern for congenital malformations was not identified is not surprising [55,56]. As noted by Gardner, “It is also possible that *B. burgdorferi* gestational infection with transplacental dissemination could cause fetal pathology simply by causing Lyme borreliosis with the same manifestations (cutaneous, musculoskeletal, neurologic, neuropsychiatric, neurocognitive and urologic) that it produces in children and adult patients…” [52]. Cases of asymptomatic, seronegative maternal infection have also been reported, with a notable case of neonatal death at 8 days, followed by autopsy, which revealed LD spirochetes in the neonate brain and heart [8]. Dattwyler and colleagues reported the case of a mother diagnosed and treated for *Borrelia* infection in her second trimester. She was asymptomatic and seronegative at the time of the birth and her child was born with neurologic dysfunction. Serological evidence of antibodies specific to *Borrelia* in the infant cerebral spinal fluid was reported [26].

Current diagnostic guidelines for Lyme disease in both North America and Europe recommend an indirect two-tier serological approach that tests for patient antibodies to the pathogen. The first tier is an enzyme immunoassay (EIA), followed by either a different EIA or a Western blot. In this case study, the mother tested positive by EIA and negative by Western blot for LD; however, a EUROIMMUN *Borrelia* line blot was later found to be positive. The child was initially negative on both the EIA and Western blot for LD, but upon repeat testing, the EIA was positive, while the Western blot remained negative. Based on serological criteria alone, the child would be considered not to have Lyme disease, yet application of advanced molecular assays and imaging methods allowed direct detection of the pathogen.

Discordance between serology and histopathological findings of *B. burgdorferi* in fetal or placental tissue has previously been reported [7,31]. One study described several cases in which maternal LD serology post-partum was negative despite histopathological findings of spirochetes in perinatal autopsy tissues [7]. Additionally, pathological examination of placentas from sixty asymptomatic women with a positive or equivocal EIA from an LD-endemic area of New York revealed spirochetes by silver stain in three of sixty (5%) placentas, with further PCR confirmation of *B. burgdorferi* in two of these three placentas [31]. Serological testing for LD in the three women with placental spirochetes showed an equivocal ELISA in all three, negative Western blots in two, and an indeterminate Western blot in one. There was no relationship between the presence of placental spirochetes and serological testing, and based on serological interpretation, two of these women would be considered not to have LD despite placental infection with the spirochete [31].

A differential diagnosis of congenitally acquired LD was not considered for the child described in this report at the time of birth or in early infancy. Even if LD had been suspected, there are currently no diagnostic criteria or treatment guidelines for congenital LD. Such guidelines do exist for other vector-borne infections known to be transmitted in utero, such as Zika virus, West Nile virus and Chagas disease [57,58,59]. In general, diagnostic approaches for congenital infections in a newborn infant may include a broad range of both indirect and direct testing methodologies including serological testing and culture and/or detection of pathogen-specific nucleic acids from tissues and body fluids [60,61].

Historical recommendations for laboratory testing for the diagnosis of congenital Lyme infection were proposed in specialty medical textbooks but never became widely known or adopted in clinical practice [52,62]. In one set of recommendations, major diagnostic criteria to confirm congenital Lyme disease included: (1) *B. burgdorferi*-specific IgM in cord blood or in the patient’s serum immediately following delivery; (2) culture of spirochetes from the placenta or the newborn; or (3) histological identification of *Borrelia* in infant tissues utilizing *Borrelia*-specific immunofluorescent techniques [52]. Separate recommendations from an infectious disease pediatrician advised evaluation of any infant with suspected congenital LD with serologic testing for both *B. burgdorferi* IgM and IgG ELISA and IgM and IgG Western blot on paired maternal and cord blood at delivery, along with infant’s blood and preferably cerebrospinal fluid after birth, and if possible, culture and PCR testing for *B. burgdorferi* should be performed on these samples [52]. In addition, a full histopathological examination of any placenta, miscarriage, stillbirth or perinatal death from a pregnancy impacted by LD should be undertaken, which utilizes testing such as silver and *B. burgdorferi*-specific antibody stains, culture or PCR [52].

Several early case reports documenting histopathological findings of *B. burgdorferi* identified in either fetal/infant or placental tissues revealed a lack of or minimal accompanying inflammation [4,6,7,9]. A 2011 report from the Czech Republic documented evidence of *Borrelia* detected in placental tissues by PCR, culture and electron microscopy from women who had been treated with penicillin for LD in the first trimester of pregnancy, noting placental inflammation was minimal [29].

In this study, the child’s infection could be due to either vector-borne transmission *B. burgdorferi* sensu stricto strain specific to North America of the mother’s infection, congenital transmission, or both. A potential role of other infections in promoting transplacental transmission, such as the uncharacterized febrile infection of the mother during the first trimester of pregnancy, is something that warrants further investigation. As both mother and child present with the same strain of *B. burgdorferi*—specifically, the widely distributed species common to their region of residence, *B. burgdorferi* s.s. *ospC* type A, and given the presence of *B. burgdorferi* identified in the placental tissues through both immunostaining and DNA analysis, vertical transmission in this case is a plausible, and the most parsimonious, explanation. The findings in this study support the need for further research into alternative, non-vector routes of *Borrelia* transmission. This topic deserves attention, as the results heighten the diagnostic complexity of managing Lyme disease if it is both a vector-borne and vertically transmitted disease.

## Figures and Tables

**Table 1:** Summary of samples and findings.

Subject	Tissues Tested	Methods Applied	Targeted Genes/Loci	Results Obtained
adult	urine ^4^	cultivation		negative
	periodontal swab ^4^	cultivation		negative
	vaginal swab ^4^	cultivation		positive
		PCR	*osp A*, *flaB*, *16S-23S ITR*	*ospA*
	blood ^1^	cultivation		negative
		PCR	*fla B*, *osp C*, *p66*, *recG*, *rpl B*, *uvrA*	*fla B*, *p66*
	placenta ^2,3^	indirect immunofluorescence		positive
		PCR	*flaB*, *P13*	*flaB*, *P13*
child	urine ^4^	cultivation		positive
		PCR	*osp A*, *flaB*, *16S-23S ITR*	*ospA*
	blood ^1^	cultivation		negative
		PCR	*fla B*, *osp C*, *p66*, *recG*, *rpl B*, *uvrA*	*fla B*, *osp C*, *p66*, *rpl B*, *uvrA*

^1^—tests conducted by European team; ^2,3^—tests conducted by US team; ^4^—tests conducted by Canadian team.

## Data Availability

The original contributions presented in the study are included in the article/Supplementary Materials, further inquiries can be directed to the corresponding author.

## Supplementary Materials

The following supporting information can be downloaded at: https://www.mdpi.com/article/10.3390/microorganisms14020406/s1, Figure S1: Archival tissue blocks obtained for research purposes from the hospital where the child was born. Multiple sections were cut from each block, collected in 1.5 ml tubes, and deparaffinized for immunostaining and nucleic acid amplification. Figure S2: No primary control image. A duplicate sample from block D was stained with DAPI only. A single section revealed no signal in the green channel. Nikon Ti2 confocal microscope (PLAN APO λD 40× objective). Figure S3: Higher resolution single plane merged channels image of an ir-B. burgdorferi stained spirochete in placental tissue from Block D (from Figure 2). Nikon Ti2 confocal microscope (PLAN APO λD 60× OIL OF N 25 DIC N2 objective). Table S1: PCR primers used for amplification of selected Borrelia loci (CA). Table S2: PCR primers used for amplification of selected Borrelia loci (CZ). Table S3: PCR primers used for amplification of selected Borrelia loci (USA). References [44,63,64,65,66,67,68,69] are cited in the supplementary materials.

## Abbreviations

The following abbreviations are used in this manuscript:

LD	Lyme disease
EIA	Enzyme immunoassay
BSK	Barbour–Stoenner–Kelly
FFPE	Formalin-fixed paraffin-embedded
