# Supplementary material for: Advanced Molecular and Microscopic Diagnostics Suggest Congenital Borrelia Transmission: A Case Report
Source: Microorganisms. 2026 Feb 9;14(2):406. doi: 10.3390/microorganisms14020406 (PMC12943057; doi:10.3390/microorganisms14020406)
Supplement: Supplementary file 1 [file microorganisms-14-00406-s001.zip › microorganisms-3999366-supplementary.pdf]

SUPPLEMENTARY MATERIALS [Case report submission 3999366]

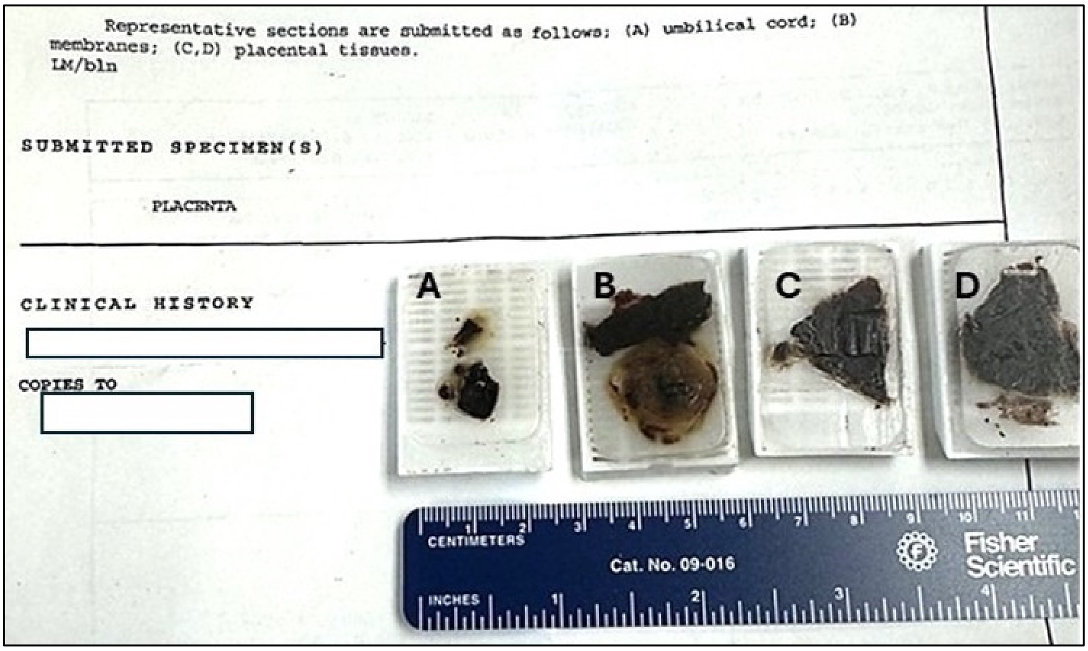

Supplemental Figure S1. Archival tissue blocks obtained for research purposes from the hospital where the child was born. Multiple sections were cut from each block, collected in 1.5 ml tubes, and deparaffinized for immunostaining and nucleic acid amplification.

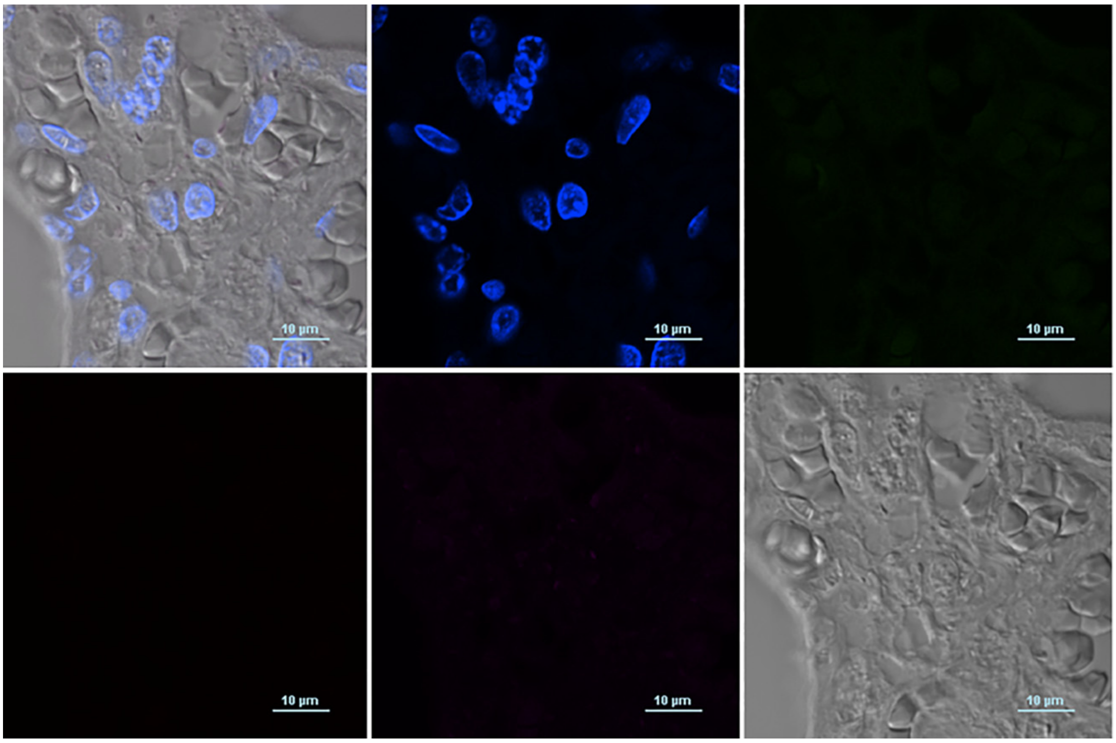

Supplemental Figure S2. No primary control image. A duplicate sample from block D was stained with DAPI only. A single section revealed no signal in the green channel. Nikon Ti2 confocal microscope (PLAN APO λD 40x objective).

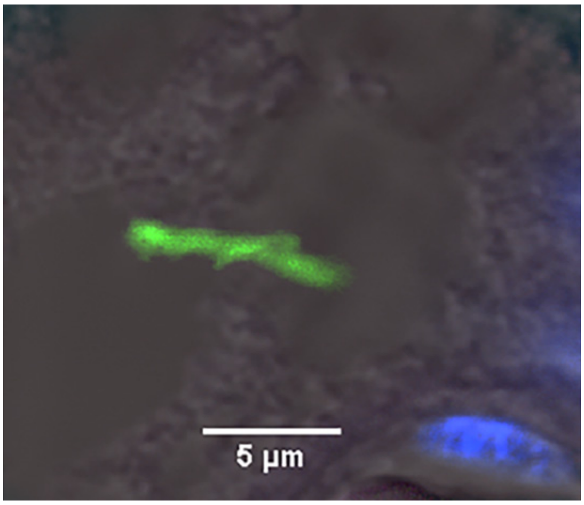

Supplemental Figure S3. Higher resolution single plane merged channels image of an ir-B. *burgdorferi* stained spirochete in placental tissue from Block D (from Figure 2). Nikon Ti2 confocal microscope (PLAN APO λD 60x OIL OF N 25 DIC N2 objective).

DNA extraction and PCR analyses of cultured samples (Canadian laboratory)

After incubation, culture samples were transferred to 1.5 ml Eppendorf tubes and centrifuged at 10,000g for 10 minutes. The supernatant was discarded into a biohazard container and later autoclaved, the pellet was resuspended in 1X PBS and stored overnight at -80ºC to lyse cells. Frozen samples were then thawed, centrifuged at 10, 000g for 10 min and supernatant discarded into a biohazard container, as above. This step may result in reduced DNA yield; any DNA in the supernatant could also be precipitated if maximum yield was needed. For the aquaplasmid reagent, DNA extraction was performed following a modified version of the manufacturer's protocol. Briefly, 200 µl of aquaplasmid solution was added to each pellet and vortexed. Samples were incubated at room temperature for 5 min followed by freezing at -20ºC for another 5 min, thawing and centrifuged at 14, 000g for 5 min, with the supernatant collected. A half volume of isopropanol was added to supernatant and mixed thoroughly, followed by centrifugation at 14, 000g for 5 min. The supernatant was discarded and the pellet was rinsed with 70% ethanol. After air drying, 50 µl of 1mM Tris HCl was added to pellet and the tubes were incubated at room temperature for 5 min. Samples were centrifuged at 14, 000g for 2 min and stored at -20ºC for further molecular testing. All DNA extractions were performed in an ethanol- and UV radiation-sterilized Biological Safety Cabinet (LabGard Class II, Type A2) following standard biosafety precautions.

The PCR preparation room was separate from all molecular or microbiological workspaces. The nested PCR (nPCR) of partial *ospA* and *flaB* genes were performed as described by Wills et al. 2018. Amplification of partial 16S-23S internal transcribed region (ITR) was conducted with PCR primers set, described earlier (Bunikis et al. 2004; Table S1). Negative controls consisted water instead of template DNA. Positive controls were run at a different time to avoid contamination. PCR reactions were performed in a Labnet MultiGene OptiMax Thermal Cycler with GoTaqGreen polymerase (Promega). In the second step of nPCR, 2µl of 1st round reaction mix was used as a template. The final products were analyzed using electrophoresis on a 1.2% agarose gel in TAE buffer. A 100 bp DNA ladder RTU (FroggaBio) was used as the molecular marker.

The nPCR programs for amplification of genes encoding outer surface protein A (*ospA*) and flagellin B (*flaB*) were as follows: denaturation at 95ºC for 5 min; 40 cycles of 95ºC for 30 sec, annealing temperature (55ºC for 1st step and 58ºC for the 2nd step) for 1 min, with extension at 72ºC for 40 sec followed by 1 cycle of 72ºC for 5 min then a hold at 4ºC. For 16S-23S internal transcribed region, the amplification program for the initial reaction was 4 min at 94ºC , 35 cycles

of 94ºC for 1 min, 50ºC for 1 min, 72ºC for 1 min, followed by 72ºC for 10 min. The second round of amplification was: 5 min at 95ºC, 40 cycles of 95ºC for 30 sec, 51ºC for 30 sec, 72ºC for 30 sec, followed by 72ºC for 10 min.

Amplicons were analyzed by Sanger sequencing at Genome Quebec at McGill University. The resulting chromatograms were manually inspected for quality with Finch TV (FinchTV 1.4.0, Geospiza, Inc.; Seattle, WA, USA; http://www.geospiza.com) software. Sequences were compared to NCBI Genbank by nucleotide BLAST.

Table S1. PCR primers used for amplification of selected *Borrelia* loci (CA)

Primer name	Target gene	Sequence (5´–3´)	Amplicon size (bp)	Source
FlagB out F	*flaB*	GCATCACTTTCAGGGTCTCA	503	Wills et al. 2018
FlagB out R	*flaB*	TGGGGAACTTGATTAGCCTG		
FlagB in F	*flaB*	CTTTAAGAGTTCATGTTGGAG	447	Wills et al. 2018
FlagB in R	*flaB*	TCATTGCCATTGCAGATTGT		
OspA out F	*ospA*	CTTGAAGTTTTCAAAGAAGAT	487	Wills et al. 2018
OspA out R	*ospA*	CAACTGCTGACCCCTCTAAT		
OspA in F	*ospA*	ACAAGAGCAGACGGAACCAG	350	Wills et al. 2018
OspA in R	*ospA*	TTGGTGCCATTTGAGTCGTA		
Rrs	*16S-23S ITR*	GTATGTTTAGTGAGGGGGGTG	441	Bunikis et. al 2004
Rrl	*16S-23S ITR*	GGATCATAGCTCAGGTGGTTAG		

DNA extraction and PCR analyses of cultured samples (European laboratory)

All cultures without *Borrelia* growth were centrifuged at 13, 200 g for 20 minutes, and the resulting pellets were used for genomic DNA extraction. Genomic DNA was extracted using the Qiagen DNeasy Blood and Tissue Kit (Qiagen, Germany) strictly following the manufacturer's protocol. Purified genomic DNA was further used for PCR analyses with different sets of PCR primers. DNA purification, PCR amplification, and post-amplification analyses were performed in separate areas with all necessary precautions to prevent contamination. The presence of *Borrelia* DNA in

the samples was assessed by PCR amplification of partial genes encoding *p66* (Bunikis et al., 2004), outer surface protein C (*ospC*) (Bunikis et al., 2004) , *flagellin* (Clark et al., 2005), and three housekeeping genes – *recG, rplB*, and *uvrA* – using the MLST protocol previously described by Margos (Margos et al., 2008). To prevent inhibition of the reactions by a possible excess of human DNA, all PCRs were performed in two steps: the first (spacer) and the second (nested). Reaction mix of the first amplification was used as the template for the second reaction. All PCR reactions were carried out in a final volume of 20 µl using 2x HotStarTaq Plus Master Mix (Qiagen, Germany). Amplicons were visualized by electrophoresis in a 1% agarose gel (1× TAE, pH 8.0) stained with SYBR Gold DNA gel stain (Invitrogen, USA). In all cases, a reaction mix with sterile ddH_2O instead of template DNA was used as a negative control. Purified DNA from *Borrelia carolinensis* was used as a positive control in all PCR reactions. All PCR products of the expected sizes were excised from the agarose gels, purified using ULTRAFREE centrifugal filter units for DNA extraction from agarose (Millipore, USA), and sequenced in both directions using the same primers as for PCR. Sequence analysis was performed by SEQme (SEQme s.r.o., Czech Republic). All sequences were compared to those available in the GenBank dataset by Basic Local Alignment Tool (BLAST) analysis.

The fragment of *p66* gene was amplified by spacer/nested PCR using the previously described primers and protocol by Bunikis et al., 2004 (Table S2). The conditions of both reactions were: 95 °C for 30 sec, 50 °C for 30 sec, and 72 °C for 60 sec except for the number of PCR cycles, – 30 in the spacer step and 25 in nested step.

The fragment of *osp C* gene was amplified by spacer/nested PCR using the same protocol by Bunikis et al., 2004. Amplification of partial *flagellin B* gene was done with PCR primers described by Clark et al., 2005. The conditions of both reactions were identical: 30 cycles at 95 °C for 30 sec, 52 °C for 30 sec, and 72 °C for 30 sec, except the annealing temperature that was 52 and 55 °C for the spacer and nested step of PCR respectively.

The PCR conditions for *rplB* and *uvrA* housekeeping genes were as follows: for the first set of cycles, touchdown PCR was used with annealing temperatures starting from 55ºC and decreasing 1ºC each cycle. Specific conditions were 95ºC for 15 min, 94ºC for 30 sec, annealing temperature from 55ºC to 48ºC for 30 sec, and an extension step of 72ºC for 60 sec. An additional 20 cycles were run at 94ºC for 30 sec, annealing temperature of 48ºC for 30 sec, and extension at 72ºC for 60 sec. After a final extension step for 5 min at 72ºC, the samples were kept at 14ºC until further

analysis. The conditions for the second set of 35 cycles were 95ºC for 7 min, 94ºC for 30 sec, 50 ºC for 30 sec, 72ºC for 60 sec. After a final extension step for 5 min at 72ºC, the samples were kept at 14ºC.

For *recG*, the PCR conditions for the first set of cycles were 95ºC for 15 min, followed by 30 cycles of 94ºC for 30 sec, 55ºC for 30 sec, 72ºC for 60 sec, and extension at 72ºC for 5 min. The conditions for the second set of cycles were identical.

Table S2. PCR primers used for amplification of selected *Borrelia* loci (CZ)

Primer name	Target gene	Sequence (5´–3´)	Amplicon size (bp)	Source
spacer P66 F	*P66*	GATTTTTCTATATTTGGACACAT	724	Bunikis et al. (2004)
spacer P66 R	*P66*	TGTAAATCTTATTAGTTTTTCAAG		
nested P66 Fn	*P66*	CAAAAAAGAAACACCCTCAGATCC	655	Bunikis et al. (2004)
nested p66 Rn	*P66*	CCTGTTTTTAAATAAATTTTTGTAGCATC		
osp C F1	*osp C*	ATGAAAAAGAATACATTAAGTGC	657	Bunikis et al. (2004)
osp C R1	*osp C*	ATTAATCTTATAATATTGATTTTAATTA AGG		
osp C F2	*osp C*	TATTAATGACTTTATTTTTATTTATATCT	617	Bunikis et al. (2004)
osp C R2	*osp C*	TTGATTTTAATTAAGGTTTTTTTGG		
fla F1	*flaB*	AARGAATTGGCAGTTCAATC	465	Clark et al. (2005)
fla R1	*flaB*	GCATTTTCWATTTTAGCAAGTGATG		
fla F2	*flaB*	ACATATTCAGATGCAGACAGAGGTTCTA	388	Clark et al. (2005)
fla R2	*flaB*	GAAGGTGCTGTAGCAGGTGCTGGCTGT		
rplB outer F	*rplB*	TGGGTATTAAGACTTATAAGC	758	Margos et al. (2008)
rplB outer R	*rplB*	GCTGTCCCCAAGGAGACA		
rplB inner F	*rplB*	CGCTATAAGACGACTTTATC	720	Margos et al. (2008)
rplB inner R	*rplB*	GCTGTCCCCAAGGAGACA		
recG outer F	*recG*	CCCTTGTTGCCTTGCTTTC	804	Margos et al. (2008)
recG outer R	*recG*	GAAAGTCCAAAACGCTCAG		

recG inner F	*recG*	CTTTAATTGAAGCTGGATATC	741	Margos et al. (2008)
recG inner R	*recG*	CAAGTTGCATTTGGACAATC		
uvrA outer F	*uvrA*	TGGGTATTAAGACTTATAAGC	910	Margos et al. (2008)
uvrA outer R	*uvrA*	GCTGTCCCCAAGGAGACA		
uvrA inner F	*uvrA*	CGCTATAAGACGACTTTATC	677	Margos et al. (2008)
uvrA inner R	*uvrA*	GCTGTCCCCAAGGAGACA		

DNA extraction DNA and PCR analyses from formalin-fixed tissue (USA laboratory)

The brief step-by-step procedure for de-paraffinization of embedded tissues is:

1. Incubation of slides for 5 minutes in clean xylene;* repeat once.
2. Incubation of slides for 5 minutes in 100% ethanol; repeat once.
3. Incubation of slides for 5 minutes in [95% ethanol: 5% water]; repeat once.
4. Incubation of slides for 5 minutes in [85% ethanol: 15% water].
5. Incubation of slides for 5 minutes in [70% ethanol: 30% water].
6. Incubation of slides for 5 minutes in [50% ethanol: 50% water].
7. Incubation of slides for 5 minutes in [30% ethanol: 70% water].
8. Incubation of slides for 5 minutes in ultrapure water; repeat once.

After de-paraffinization, the Qiagen protocol was used strictly as recommended. To extract the DNA from the first column, 350 µl of AW1 AllPrep buffer was added to the column and spun for 1 minute at 13,200 rpm. Twenty microliters of proteinase K were added to 60 µl of AW1, added to the column and incubated at room temperature for 5 minutes. After the incubation 350 µl of AW1 was added to the column, followed by 1 minute spin at 13,200 rpm. After the wash with 500 µl of AW2 buffer elution of purified DNA was done by adding of 50 µl of ddH_2O to the column, brief incubation at room temperature and spun at 13,200 rpm. Elution step was repeated twice. Purified DNA was used as template in PCR as described by Ericson et all., 2024 and Clark et al., 2005. Negative controls of water only were used for each set of primers and the water-only reaction from the first round of nested PCR was then used again as another negative control in the second

round of PCR for flaB. The p13 amplification included a water negative control and was not a nested protocol.

Table S3. PCR primers used for amplification of selected *Borrelia* loci (USA)

Primer name	Target gene	Sequence (5′–3′)	Amplicon size (bp)	Source
P13 forward	*P13*	ATGAAACTAGCAAGCAAGATCCTAT TGTACC	135	Ericson et al. (2024)
P13 reverse	*P13*	GCCCTATACCAACCGCATCAAATC		
Fla outF	*flaB*	AARGAATTGGCAGTTCAATC	465	Clark et al. (2005)
Fla outR	*flaB*	GCATTTTCWATTTTAGCAAGTGATG		
flaB inner F	*flaB**	TAGAGCAACTTACAGACGAAATTA	384	this study; designed by USA team
flaB inner R	*flaB**	TTGTAACATTAACAGGAGAAT TAACTC		

References

44. Ericson, M.E.; Mozayeni, B.R.; Radovsky, L.; Bemis, L.T. Bartonella- and borrelia-related disease presenting as a neurological condition revealing the need for better diagnostics. *Microorganisms* **2024**, *12*, 209. doi: 10.3390/microorganisms12010209

63. Miklossy, J.; Kasas, S.; Zurn, A.D.; McCall, S.; Yu, S.; McGeer, PL. Persisting atypical and cystic forms of Borrelia burgdorferi and local inflammation in Lyme neuroborreliosis. *J Neuroinflammation.* **2008**, 5,40. doi: 10.1186/1742-2094-5-40.

64. Sloupenska K.; Koubkova B.; Horak P.; Dolezilkova J.; Hutyrova B.; Racansky M.; Miklusova M.; Mares J.; Raska M.; Krupka M.; Antigenicity and immunogenicity of different morphological forms of Borrelia burgdorferi sensu lato spirochetes. *Sci Rep*. **2024**, 14(1),4014. doi: 10.1038/s41598-024-54505-y.

65. Wills, M.K.B.; Kirby, A.M.; Lloyd V.K. Detecting the Lyme disease spirochete, *Borrelia burgdorferi*, in ticks using nested PCR. *J. Vis. Exp.* **2018**, *4,* 56471. doi: 10.3791/56471.

66. Bunikis, J.; Garpmo, U.; Tsao, J.; Berglund, J.; Fish, D.; Barbour, A.G. Sequence typing reveals extensive strain diversity of the Lyme borreliosis agents *Borrelia burgdorferi* in North America and *Borrelia afzelii* in Europe. *Microbiology* **2004**,*150*, 1741–1755.

67. FinchTV 1.4.0 Geospiza, Inc.; Seattle, WA, USA; http://www.geospiza.com

68. Clark, K.; Hendricks, A.; Burge, D. Molecular identification and analysis of *Borrelia burgdorferi* sensu lato in lizards in the Southeastern United States. ASM Journals *AEM* **2005**, *71*, 5. https://doi.org/10.1128/AEM.71.5.2616-2625.2005

69. Margos, G.; Gatewood, A.G.; Aanensen, D.M.; Hanincová, K.; Terekhova, D.; Vollmer, S.A.; Cornet, M.; Piesman, J.; Donaghy, M.; Bormane, A.; Hurn, M.A.; Feil, E.J.; Fish, D.; Casjens, S. MLST of housekeeping genes captures geographic population structure and suggests a European origin of Borrelia burgdorferi. *Proc. Natl. Acad. Sci. USA* **2008**, *105*, 8730–8735. https://doi.org/10.1073/pnas.0800323105

SEQUENCES obtained in this study.

Canada

Sequences for adult subject sample “vaginal swab” (Canada-211 nt). Target: gene encoding outer surface protein A (*ospA*):

AAAGAGGTTTTAAAAGGCTATGTTCTTGAAGGAACTCTAACTGCTGAAAAAACAACATTGGTGGTTAA
AGAAGGAACTGTTACTTTAAGCAAAAATATTTCAAAATCTGGGGAAGTTTCAGTTGAACTTAATGACA
CTGACAGTAGTGCTGCTACTAAAAAAACTGCAGCTTGGAATTCAGGCACTTCAACTTTAACAATTACT
GTAAACA

Sequences for child subject sample “urine” (Canada-221 nt). Target: gene encoding outer surface protein A (*ospA)*:
TAAAGAGGTTTTAAAAGGCTATGTTCTTGAAGGAACTCTAACTGCTGAAAAAACAACATTGGTGGTTA
AAGAAGGAACTGTTACTTTAAGCAAAAATATTTCAAAATCTGGGGAAGTTTCAGTTGAACTTAATGAC
ACTGACAGTAGTGCTGCTACTAAAAAAACTGCAGCTTGGAATTCAGGCACTTCAACTTTAACAATTAC
TGTAAACAGTAAAAAAA

USA

Sequences for adult subject sample “placenta” (USA-384 nt). Target: gene encoding *flagellin* B protein -original
TTGTAACATTAACAGGAGAATTAACTCCGCCTTGAGAAGGTGCTGTAGCGGGTGCTGGCTGTTGAGCT
CCTTCCTGTCGAACACCCTCTTGAACCGGTGCAGCCTGAGCAGTTTGAGCTCCCTCACCAGAGAAAAG
ATTTGCAACATTAGCTGCATAAATATTTACAGCAATAGCTTCATCTTGGTTTGCTCCAACATGAACTCT
TAAAGTCCAAGACGCTTGAGACCCTGAAAGTGATGCTGGTGTGTTAATTTTTGCAGGCTGCATTCCAA
GCTCTTCAGCTGTTCTTACATTTTGAGAAGCAGATTTGTTTGATAACATGTGCATTTGGTTATATTGAGC
TTGATCAGCAATTCTATTAATTTCGTCTGTAAGTTGCTCTA

Reverse complement *flaB* (used for alignment)
TAGAGCAACTTACAGACGAAATTAATAGAATTGCTGATCAAGCTCAATATAACCAAATGCACATGTTA
TCAAACAAATCTGCTTCTCAAAATGTAAGAACAGCTGAAGAGCTTGGAATGCAGCCTGCAAAAATTAA
CACACCAGCATCACTTTCAGGGTCTCAAGCGTCTTGGACTTTAAGAGTTCATGTTGGAGCAAACCAAG
ATGAAGCTATTGCTGTAAATATTTATGCAGCTAATGTTGCAAATCTTTTCTCTGGTGAGGGAGCTCAAA
CTGCTCAGGCTGCACCGGTTCAAGAGGGTGTTCGACAGGAAGGAGCTCAACAGCCAGCACCCGCTAC
AGCACCTTCTCAAGGCGGAGTTAATTCTCCTGTTAATGTTACAA

(translated protein seq):
EQLTDEINRIADQAQYNQMHMLSNKSASQNVRTAEELGMQPAKINTPASLSGSQASWTLRVHVGANQDE
AIAVNIYAANVANLFSGEGAQTAQAAPVQEGVRQEGAQQPAPATAPSQGGVNSPVNVT

Sequences for adult subject sample “placenta” (USA-136 nt). Target: gene encoding an integral membrane protein P13
GCCCTATACCAACCGCATCAAATCCAAGAATAAGAGAACCTCCAAGAATATCTCCTTGAGCAAAGGA
GCCTATTCCAAACCCTAAAAAAAGGTTCAATAAAAATGGTACAATAGGATCTTGCTTGCTAGTTTCAT
A

Europe

Sequence obtained from adult subject "blood" sample (CZ-236 nt). Target: gene encoding outer membrane protein p66:
TGCAGAAACACCTTTTGAATTAAATTTTGGCTTGTCAGGAGCCTATGGAAACGAGAC**A**TTCAATAATT
CATCAATAACATACTCTTTAAAAGATAAATCCGTAGTTGGCAACGATTTATTGAGCCCAACTTTATCAA
ATTCTGCAATTTTAGCATCTTTTGGAGCTAAATATAAGCTTGGATtAACAAAAATAAACGATAAAAATA
CCTATCTTATTTTGCAAATGGGAACTGATT

Sequences obtained from adult subject sample "blood" (CZ-390nt).Target: gene encoding *flagellin* B protein:
ACATATTCAGATGCAGACAGAGGTTCTATACAAATTGAAATAGAGCAACTTACAGACGAAATTAATAG
AATTGCTGATCAAGCTCAATATAACCAAATGCACATGTTATCAAACAAATCTGCTTCTCAAAATGTAA
GAACAGCTGAAGAGCTTGGAATGCAGCCTGCAAAAATTAACACACCAGCATCGCTTTCAGGGTCTCAA
GCGTCTTGGACTTTAAGAGTTCATGTTGGAGCAAACCAAGATGAAACTATTGCTGTAAATATTTATGC
AGCTAATGTTGCAAATCTTTTCTCTGGTGAGGGAGCTCAAACTGCTCAGGCTGCACCGGTTCAAGAGG
GTGTTCAACAGGAAGGAGCTCAACAGCCAGCACCTGCTACAGCACCTTCA

(translated protein seq):
TYSDADRGSIQIEIEQLTDEINRIADQAQYNQMHMLSNKSASQNVRTAEELGMQPAKINTPASLSGSQASW
TLRVHVGANQDETIAVNIYAANVANLFSGEGAQTAQAAPVQEGVQQEGAQQPAPATAPS

Alignment of nucleotide sequences of flagellin B from CZ "blood" sample (Query) with USA- "placenta" sample (subject 1)

```
Sequence ID: Query_4553209  Length: 384  Number of Matches: 1

Range 1: 1 to 349  Graphics                    ▼ Next Match  ▲ Previous Match

Score           Expect  Identities       Gaps        Strand
623 bits(337)   0.0     345/349(99%)     0/349(0%)   Plus/Plus

Query  41   TAGAGCAACTTACAGACGAAATTAATAGAATTGCTGATCAAGCTCAATATAACCAAATGC  100
            ||||||||||||||||||||||||||||||||||||||||||||||||||||||||||||
Sbjct  1    TAGAGCAACTTACAGACGAAATTAATAGAATTGCTGATCAAGCTCAATATAACCAAATGC  60

Query  101  ACATGTTATCAAACAAATCTGCTTCTCAAAATGTAAGAACAGCTGAAGAGCTTGGAATGC  160
            ||||||||||||||||||||||||||||||||||||||||||||||||||||||||||||
Sbjct  61   ACATGTTATCAAACAAATCTGCTTCTCAAAATGTAAGAACAGCTGAAGAGCTTGGAATGC  120

Query  161  AGCCTGCAAAAATTAACACACCAGCATCGCTTTCAGGGTCTCAAGCGTCTTGGACTTTAA  220
            |||||||||||||||||||||||||||| |||||||||||||||||||||||||||||||
Sbjct  121  AGCCTGCAAAAATTAACACACCAGCATCACTTTCAGGGTCTCAAGCGTCTTGGACTTTAA  180

Query  221  GAGTTCATGTTGGAGCAAACCAAGATGAAACTATTGCTGTAAATATTTATGCAGCTAATG  280
            ||||||||||||||||||||||||||||| ||||||||||||||||||||||||||||||
Sbjct  181  GAGTTCATGTTGGAGCAAACCAAGATGAAGCTATTGCTGTAAATATTTATGCAGCTAATG  240

Query  281  TTGCAAATCTTTTCTCTGGTGAGGGAGCTCAAACTGCTCAGGCTGCACCGGTTCAAGAGG  340
            ||||||||||||||||||||||||||||||||||||||||||||||||||||||||||||
Sbjct  241  TTGCAAATCTTTTCTCTGGTGAGGGAGCTCAAACTGCTCAGGCTGCACCGGTTCAAGAGG  300

Query  341  GTGTTCAACAGGAAGGAGCTCAACAGCCAGCACCTGCTACAGCACCTTC  389
            |||||| ||||||||||||||||||||||||||| ||||||||||||||
Sbjct  301  GTGTTCGACAGGAAGGAGCTCAACAGCCAGCACCCGCTACAGCACCTTC  349
```

Sequence ID: Query_866401 Length: **127** Number of Matches: **1**

Range 1: 1 to 116 Graphics ▼ Next Match ▲ Previous Match

Score	Expect	Method	Identities	Positives	Gaps
229 bits(585)	2e-84	Compositional matrix adjust.	114/116(98%)	115/116(99%)	0/116(0%)

```
Query  15   EQLTDEINRIADQAQYNQMHMLSNKSASQNVRTAEELGMQPAKINTPASLSGSQASWTLR  74
            EQLTDEINRIADQAQYNQMHMLSNKSASQNVRTAEELGMQPAKINTPASLSGSQASWTLR
Sbjct  1    EQLTDEINRIADQAQYNQMHMLSNKSASQNVRTAEELGMQPAKINTPASLSGSQASWTLR  60

Query  75   VHVGANQDETIAVNIYAANVANLFSGEGAQTAQAAPVQEGVQQEGAQQPAPATAPS  130
            VHVGANQDE IAVNIYAANVANLFSGEGAQTAQAAPVQEGV+QEGAQQPAPATAPS
Sbjct  61   VHVGANQDEAIAVNIYAANVANLFSGEGAQTAQAAPVQEGVRQEGAQQPAPATAPS  116
```

Sequences obtained from the child subject sample “blood” (CZ-389nt).Target: gene encoding *flagellin* B protein
ACATATTCAGATGCAGACAGAGGTTCTATACAAATTGAAATAGAGCAACTTACAGACGAAATTAATAG
AATTGCTGATCAAGCTCAATATAACCAAATGCACATGTTATCAAACAAATCTGCTTCTCAAAATGTAA
GAACAGCTGAAGAGCTTGGAATGCAGCCTGCAAAAATTAACACACCAGCATCGCTTTCAGGGTCTCAA
GCGTCTTGGACTTTAAGAGTTCATGTTGGAGCAAACCAAGATGAAACTATTGCTGTAAATATTTATGC
AGCTAATGTTGCAAATCTTTTCTCTGGTGAGGGAGCTCAAACTGCTCAGGCTGCACCGGTTCAAGAGG
GTGTTCAACAGGAAGGAGCTCAACAGCCAGCACCTGCTACAGCACCTTC

Sequence obtained from the child subject “blood” sample (CZ-236 nt). Target: gene encoding outer membrane protein p66:
TGCAGAAACACCTTTTGAATTAAATTTTGGCTTGTCAGGAGCCTATGGAAACGAGAC**G**TTCAATAATT
CATCAATAACATACTCTTTAAAAGATAAATCCGTAGTTGGCAACGATTTATTGAGCCCAACTTTATCAA
ATTCTGCAATTTTAGCATCTTTTGGAGCTAAATATAAGCTTGGATTAACAAAAATAAACGATAAAAAT
ACCTATCTTATTTTGCAAATGGGAACTGATT

Sequences obtained from the child subject sample “blood” (CZ-618nt).Target: gene encoding outer surface protein C *(ospC):*
TATTAATGAACTTTATTTTTATTTATaTCTTGTAATAATTCAGGGAAAGATGGGaATACATCTGCAAATT
CTGCTGATGAGTCTGTTAAAGGGCCTAATCTTACAGAAATAAGTAAAAAAATTACGGATTCTAATGCG
GTTTTACTTGCTGTGAAAGAGGTTGAAGCGTTGCTGTCATCTATAGATGAAATTGCTGCTAAAGCTATT
GGTAAAAAAATACACCAAAATAATGGTTTGGATACCGAAAATAATCACAATGGATCATTGTTAGCGG
GAGCTTATGCAATATCAACCCTAATAAAACAAAAATTAGATGGATTGAAAAATGAAGGATTAAAGGA
AAAAATTGATGCGGCTAAGAAATGTTCTGAAACATTTACTAATAAATTAAAAGAAAAACACACAGAT
CTTGGTAAAGAAGGTGTTACTGATGCTGATGCAAAAGAAGCCATTTTAAAAACAAATGGTACTAAAAC
TAAAGGTGCTGAAGAACTTGGAAAATTATTTGAATCAGTAGAGGTCTTGTCAAAAGCAGCTAAAGAG
ATGCTTGCTAATTCAGTTAAAGAGCTTACAAGCCCTGTTGTGGCAGAAAGTCCAAAAAAACCTTAATT
AAAATCA

Sequences obtained from the child subject sample “blood” (CZ-712nt).Target: gene encoding *rplB* protein:
TAAGACGACTTTATCTTTTGATGATTTGAGCAAAGGGTATGATCCTTTGaAATCTTTAACAAAAGGTAA
AAAATTTAAATCGGGCAGAGATTCTTCTGGTAGGATTAGTATTAGAAGAAGAGGTGGTGGGCATAAG
AGAAAGTATAGGTTGATTGATTTTAATCGAAGAGATAAATTTAGCATTCCTGCTCGAGTTGCTTCTATT
GAATATGATCCTAATAGAAGTGCTAATATAGCTTTGCTTGTTTATAAAGATGGAGAAAAAAGGTATAT
TATTTCTCCTAAAGGCATTAAGGTTGGAGATGTTTTGGAAAGTGGTCCTAATGCTCCAATTAAAATTGG
CAATGCCTTACCTCTTGAAAATATTCCTATTGGAAGAACCGTTCACAATATTGAGCTTAATGTTGGAAA
GGGTGGACAGCTTATAAGAAGTGCTGGTGGATATGCTATGATACTTGCTTCTGACGGGAATTATGTCA
CTGTAAAATTATCATCTGGCGAGATGAGATTGATTTTTAAAAAATGTATTGCAACAATTGGTGAAATT
GGAAATGAAGATTATGCCAATATTTCTATAGGGAAAGCTGGTAAAAGTAGGTGGCTTGGTAGAARACC
CAAGGTTAGAGGTGTTGCTATGAATCCTGTTGACCATCCACATGGTGGTGGTGAAGGAAAAACTTCTG
GAGGTCGTCATCCTGTGTCTCCTTGGGGA

Sequences obtained from the child subject sample “blood” (CZ-679 nt).Target: gene encoding *uvrA* protein:

TGCTTAAATTTTTAATTGATGTTGGCCTTTCTTATTTATATTTAAATAGAATATCAGGCAGTCTATCTGG
TGGCGAGGCTCAGCGTATTAGGCTTGCTACTCAAATAGGATCAGCACTTTCGGGTGTTATTTATGTTCT
TGATGAGCCAAGTATTGGTCTTCATCAAAGAGATAATGAAAAATTAATCTCTACTCTTGTTAATCTTAA
AAATCTTGGCAATACTGTAATTGTTGTTGAACATGATGAGCAAACTTTGCGTACTGCGGACTATATTAT
TGATATGGGTCCTGGTGCTGGAATTCTTGGAGGGGAAATAGTTGCAAAGGGAGCCCTAATTGATATTT
TAAATAGCAAAAATAGTTTAACTGGTCAATATCTTAGCGGCAAGTTTAAAATAGATGTTCCAAGCTCT
AGAAGAAAGGCAGATAAGGGAGAAATTTTGCTTTTGGGCTCTAATAAAAATAATCTTAAAAATATAG
ACGTAAGTATCCCTTTGGGAGTTTTTACCGTAATAACAGGTGTTTCTGGTAGCGGAAAAAGTACTTTAC
TTAACGAGGTGTTATATCCAGCTCTTGATAGTAGATTAAAGCTTAATGAAAAGTATTGTGATGGCTTTA
AAGATATTGTTGGGTACGAAAAAATCGATAAAATTATTCAAATAAATCAAAAACCAATAGG
